# Effect of hydraulic retention time on microbial community structure in wastewater treatment electro‐bioreactors

**DOI:** 10.1002/mbo3.590

**Published:** 2018-03-24

**Authors:** Nancy A. ElNaker, Ahmed F. Yousef, Shadi W. Hasan

**Affiliations:** ^1^ Department of Chemical Engineering Khalifa University of Science and Technology Abu Dhabi United Arab Emirates; ^2^ Department of Physics Faculty of Science Ain Shams University Cairo Egypt; ^3^ Department of Chemistry Khalifa University of Science and Technology Abu Dhabi United Arab Emirates

**Keywords:** DNA sequencing, electric field, hydraulic retention time, microbial community, Wastewater

## Abstract

The impact of hydraulic retention time (HRT) on the performance and microbial community structure of control and electro‐bioreactors was investigated. Control bioreactors and electro‐bioreactors were operated at HRT ranging between 6 and 75 hr. The total bacterial counts in addition to the removal efficiency of NH_4_^+^–N, sCOD, and PO_4_^3−^–P was assessed in all the reactors tested. In addition, Illumina sequencing was performed to determine the microbial communities that developed in these reactors under each HRT condition. Phylogenetic analysis showed that *Proteobacteria* and *Bacteroidetes* were the dominant phyla in those reactors. In addition, *Nitrospira sp*. and *Pseudomonas sp*. were found to be present in electro‐bioreactors with higher relative abundance than in control bioreactors. The results presented here are the first to determine what different microbial communities in wastewater electro‐bioreactors due to the application of an electric current under different HRTs.

## 1 INTRODUCTION

Numerous wastewater treatment processes and techniques have been utilized to reduce water pollution and to improve drinking water quality. Understanding the behavior of microbial communities in biological processes has been recently the focus of many research studies. Efforts have been made to understand the functional characterization of microorganisms associated with the removal of organics, inorganics and nutrients from wastewater (Daims et al., 2015; Juretschko et al., 1998), bulking and foaming in activated sludge (Guo & Zhang, 2012), and fermentation processes (Guo & Zhang, 2012; Xia, Kong, Thomsen, & Nielsen, 2008).

The integration of electrochemical processes into membrane bioreactors (MBRs) combines biodegradation, electrochemical and membrane filtration processes into one system achieving high effluent quality as compared to conventional MBRs and activated sludge processes (Ensano et al., 2016). New and cheaper developments have suggested that the continuous and intermittent application of a direct current (DC) field has proven to enhance membrane filterability and is similarly effective in controlling membrane fouling (Akamatsu, Lu, Sugawara, & Nakao, 2010; Akamatsu et al., 2012; Liu, Liu, Gao, & Yang, 2012; Liu et al., 2014). The intermittent application also minimizes the direct exposure of bacteria to the electric field, potentially reducing the negative effects to the microbial community (Akamatsu et al., 2010; Bani‐Melhem & Elektorowicz, 2010; Hasan, Elektorowicz, & Oleszkiewicz, 2014; Liu et al., 2012). We have previously reported that the application of DC at current densities of 5 and 10 Am^−2^ in electro‐bioreactors led to enhancement in bioreactor performance as well as an increase in total bacterial counts and an apparent change in the microbial community structure (Zeyoudi et al., 2015). The performance and microbial community structure in an electro‐bioreactor can be influenced by many operating parameters such as nutrient content, anoxic/aerobic phase fraction, salinity, solid retention time (SRT) and hydraulic retention time (HRT) (Fontenot, Bonvillain, Kilgen, & Boopathy, 2007). Among the above‐mentioned parameters, HRT is regarded as one of the important operating parameters affecting the performance and microbial community of a bioreactor (Wang, Peng, & Stephenson, 2009). The effects of HRT and sludge properties (SRT and MLSS “Mixed Liquor Suspended Solids”) in wastewater treatment using electrically enhanced MBR were previously explored by our group, where it was shown that increasing HRT is correlated with a reduction in COD, nitrogen and phosphorous content due to more exposure time of reactor content to biodegradation and electrocoagulation (Giwa & Hasan, 2015). Other studies reported the effects of HRT on the performance and microbial community of laboratory up flow anaerobic sludge blanket (UASB) reactor treating synthetic wastewater containing trichloroethylene (TCE) (Zhang, Wang, Hu, & Li, 2015). Their results showed that the percentages of bacterial groups in each sample varied depending on the HRTs at different taxonomic levels where the potential function of dominant genera also showed and revealed the whole bacterial evolution of the biodegradation of TCE. High‐throughput sequencing technologies have significantly improved researchers’ ability to investigate microbial communities in various municipal and industrial WWTPs (Ibarbalz, Figuerola, & Erijman, 2013; Ma et al., 2015; McLellan, Huse, Mueller‐Spitz, Andreishcheva, & Sogin, 2010; Roesch et al., 2007; Zhang et al., 2012; Zhang et al., 2015). Indeed, Illumina MiSeq has been successfully used to study various environmental and industrial systems in recent years (Caporaso et al., 2012; Gibson et al., 2014; Li et al., 2014; Liang et al., 2014). In this study, activated sludge samples were collected from the MBR plant at Masdar city and were analyzed using Illumina MiSeq after being used as inoculum in batch electro‐bioreactors operated under different conditions for twenty‐four hours. The sequencing data was analyzed using Quantitative Insights Into Microbial Ecology (QIIME^™^) in order to elucidate alpha (α) and beta (β) diversity present in the different test reactors. Our data is the first to investigate the effect of HRT on the performance and microbial community structure of wastewater electro‐bioreactors. The major objectives of this study were (a) to investigate the effect of HRT on the performance of bioreactor and electro‐bioreactor; (b) to illustrate the effect of HRT on microbial community structure and function; (c) to differentiate between the effects of short HRT and long HRT linking the performance and functional bacterial groups in both bioreactors and electro‐bioreactors.

## 2 EXPERIMENTAL PROCEDURES

### 2.1 Electro‐bioreactor experimental design

**Figure 1:**
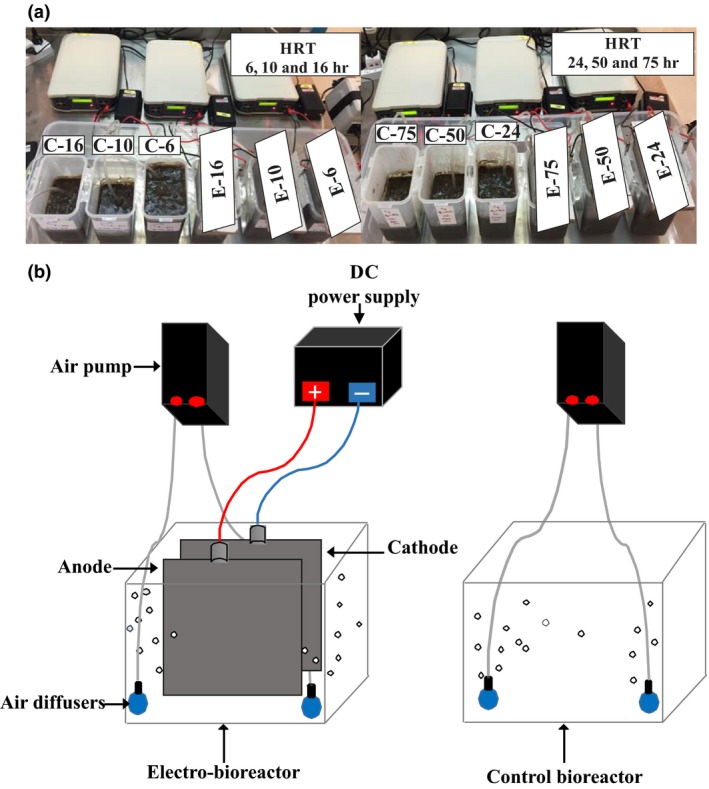
(a) Photograph and (b) schematic diagram of the experimental setup of control bioreactor and electro‐bioreactors operated at CD of 3 Am^−2^ under different HRTs: Short HRTs (6, 10, 16) long HRTs (24, 50 and 75 hr)

The experiments in this research study were conducted to evaluate the microbial community under current density of 3 Am^−2^ and HRT of 6, 10, 16, 24, 50 and 75 hr. The purpose of this design was to determine the operating conditions that favor an optimal effluent quality and microbial community. The selected range of 3 Am^−2^ was according to a previous study by our group (Zeyoudi et al., 2015), whereas the range of selected HRTs was in accordance with industrial scale MBR wastewater treatment plants (Tchobanoglous, Metcalf & Eddy, Burton, & Stensel, 2003). All bioreactors were fed with synthetic wastewater (0.2% Glucose, 1.5 mmol/L ammonium sulfate, 270 μmol/L potassium phosphate, 160 μmol/L magnesium sulfate, 20 μmol/L manganese sulfate, 1.47 μmol/L iron (III) chloride, 20 μmol/L calcium chloride, 330 μmol/L potassium chloride, 300 μmol/L sodium bicarbonate). Fresh activated sludge was collected from Masdar city's MBR wastewater treatment plant (Abu Dhabi – UAE) and used immediately to avoid any changes in its physiochemical and microbiological characteristics. The system used for this study was aerobic batch electro‐bioreactors containing sludge and synthetic wastewater prepared in the laboratory. Each bioreactor was operated under a different HRT, which was calculated as per the effective volume of the reactor (V) and the feed (i.e., synthetic wastewater) flow rate (Q) according to HRT = V/Q. For example, 6 hr HRT was calculated via adding 1200 ml of wastewater per day to 300 ml of sludge sample in a batch mode. The same method was followed to adjust the other HRTs. A total of 12 (as shown in Figure 1a) bio‐ and electro‐bioreactors were operated in parallel to ensure consistency in all experiments though which the same sludge having the same physiochemical and biological characteristics was used. The electrodes used in all experiments consisted of rectangular sheets of perforated aluminum with 75% opening as the anode, and stainless steel as the cathode spaced 5 cm apart. The effective surface area was calculated in each operating condition depending on the volume of the sample, by multiplying the width by the immersed length of the anode in the bioreactor. Aeration was provided via one small ceramic ball air stone diffusers (2‐inch diameter) placed in each reactor, connected to air pumps placed at the bottom in order to provide oxygen necessary (>2 mg L^−1^) for aerobic microbial growth and to ensure homogenous mixing, thus no loss due to evaporation was assumed to occur. A reference control bioreactor (0 Am^−2^) was used in all conditions and had no electrodes.

### 2.2 Synthetic wastewater and sludge characteristics

Synthetic wastewater and sludge dissolved oxygen (DO in mg L^−1^), pH, temperature (T in °C) and electrical conductivity (EC in μS cm^−1^) were analyzed using a HACH HQ40d single‐input multi‐meter probe (Hach Company, Loveland, CO, USA). Influent and supernatant soluble chemical oxygen demand (sCOD), phosphorous (PO_4_^3−^–P), ammonium (NH_4_^+^‐N) and nitrate (NO_3_^−^–N) were measured using HACH vials LCK 314‐1014, LCK 348‐349, LCK 303‐304 and LCK 339‐340, respectively, according to manufacturer's instructions. Table 1 shows initial characteristics of synthetic wastewater and sludge samples. At the end of each experiment, a 50 ml sample was collected from each reactor cell and centrifuged for 15 min at 3,885*g*. The supernatant was then used for determining sCOD, PO_4_^3−^–P, NH_4_^+^–N and NO_3_^−^–N. At the end of each experiment, oxygen uptake rate (OUR) was measured after cutting off aeration in all bioreactors through which the DO probe (Hach HQ40d) was immersed in the sludge and DO depletion was monitored by taking a reading every minute over a period of 15 min. The slope of the DO vs time plot represents the OUR (in mgO_2_ L^−1^ hr^−1^).

**Table 1:** Initial characteristics of synthetic wastewater and sludge samples

Parameter	Synthetic wastewater	Sludge
sCOD, mg L^−1^	2309 ± 30	–
PO_4_^3−^–P, mg L^−1^	7.1 ± 1.5	–
NO_3_^−^–N, mg L^−1^	0.18 ± 0.02	–
NH_4_^+^–N, mg L^−1^	42.5 ± 3.5	–
Mixed liquor suspended solids (MLSS), mg L^−1^	–	8134 ± 30
pH	7.1 ± 0.3	8.6 ± 0.4
T, °C	22.5 ± 2.1	23.4 ± 1.6
EC, μS cm^−1^	548 ± 10	674 ± 5

### 2.3 Sampling and bacterial counts

A 10 ml sample from each reactor after 24 hr was collected from the zone between the electrodes and analyzed for bacterial counts (Zeyoudi et al., 2015). The total bacterial count (TBC), determined using the plate count method (Zeyoudi et al., 2015), was done in duplicate and an average value was recorded. Briefly, 0.1 ml was taken from each sample and serially diluted from 10^−1^ to 10^−8^, followed by spreading 0.1 ml from each dilution on a Luria Broth plate (1% tryptone, 0.5% yeast extract, 0.5% NaCl, 2% agar) and incubating at 37°C for 24 hr. The TBC from each reactor was calculated and reported as colony‐forming units per milliliter (CFUmL^−1^). Results were expressed as the mean ± standard deviation. An analysis of variance was used to test the significance of the results with *p *<* *.05 determined a priori to be statistically significant.

### 2.4 Microbial community analysis

Total genomic DNA was isolated from the samples collected from all the electro‐bioreactors using the PowerSoil^®^ DNA Isolation Kit (MOBIO Laboratories Inc. Carlsbad, CA, USA). The DNA samples were sent to Macrogen Inc. (Seoul, Republic of Korea) for Illumina MiSeq sequencing. Amplicon libraries were created using the 337F and 805R 16S V3‐V4 universal primers (GACTCCTACGGGAGGCWGCAG and GACTACCAGGGTATCTAATC). The Illumina MiSeq instrument at Macrogen Inc. operates using control software v2.2 in conjunction with real‐time analysis software v1.18. Raw sequences delivered to our laboratory were analyzed by QIIME^™^ (version 1.9.1), using published bioinformatics pipelines (Kuczynski et al., 2011). Before generating any figures, we filtered the QIIME^™^ produced biom files by removing all unassigned operational taxonomic units (OTU's) and any OTU that did not at least have 5 counts in at least one of the samples tested. (α) and (β) diversity analysis were conducted to assess the microbial diversity in the serially passaged bioreactor and electro‐bioreactors (Jost, 2007). A phylogeny‐based‐weighted UniFrac distance analysis (Ju & Zhang, 2014) was used to compare between bacterial communities while BIO‐ENV trend and Spearman's rank correlation analysis was performed to investigate for electric field and operational parameters that is correlated with bacterial community diversity.

### 2.5 Operational parameters

Sludge and synthetic wastewater influent and effluent operational parameters which are the dissolved oxygen DO, (mg L^−1^), pH and electrical conductivity EC, (μS cm^−1^) were illustrated in (Table 2) showing 6 control bioreactor samples (C) and 6 electro‐bioreactor samples (E) operated at HRT of 6, 10, 16, 24, 50, and 75 hr.

**Table 2:** Operational parameters of control bioreactors samples (C‐6, C‐10, C‐16, C‐24, C‐50, and C‐75) and electro‐bioreactors samples (E‐6, E‐10, E‐16, E‐24, E‐50, and E‐75)

#Sample‐ID	C‐6	E‐6	C‐10	E‐10	C‐16	E‐16	C‐24	E‐24	C‐50	E‐50	C‐75	E‐75
DO_inf_ mg L^−1^	9.16	9.16	8.64	8.64	7.23	7.23	5.34	5.34	5.03	5.03	5.34	5.34
DO_eff_ mg L^−1^	8.67	7.19	7.28	8.25	8	7.22	7.62	5.01	6.44	3.94	3.04	6.43
pH_inf_	9.09	9.09	9.06	9.06	8.57	8.57	9.37	9.37	9.17	9.17	8.94	8.94
pH_eff_	6.41	9.03	6.75	8.25	8.88	9.25	9.11	8.87	9.02	9.03	9.2	9.24
EC_inf_ μS cm^−1^	629	629	704	704	760	760	641	641	707	707	746	746
EC_eff_ μS cm^−1^	637	462	670	494	658	522	571	442	675	627	710	582

## 3 RESULTS

### 3.1 Reactor performance and physiochemical parameters under different HRT

**Figure 2:**
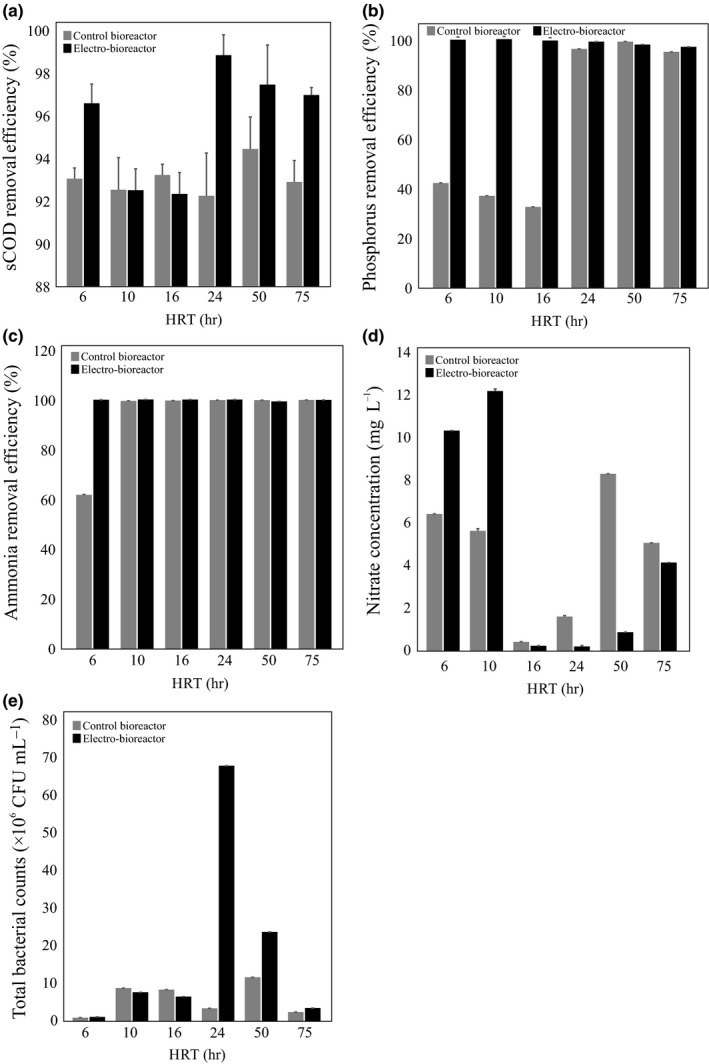
Removal efficiencies of (a) sCOD, (b) PO_4_^3−^–P and (c) NH_4_^+^–N, (d) effluent NO_3_^−^–N concentrations, and (e) total bacterial count (TBC) in control bioreactors and electro‐bioreactors under different HRTs

In order to evaluate the effect of varying HRT on bioreactor performance, the different wastewater bioreactors tested were operated at HRTs of 6, 10, 16, 24, 50 and 75 hr for 24 hr. The performance of each bioreactor was determined by measuring sCOD, PO_4_^3−^–P, NH_4_^+^–N and NO_3_^−^–N concentrations in the effluent (Figure 2). There were no differences in sCOD removal efficiencies in the control bioreactors at all HRT's tested (Figure 2a). However, the electro‐bioreactors at HRT of 24, 50, and 75 hr showed a modest improvement in sCOD removal compared to the control. On the other hand, the electro‐bioreactors operated at HRT of 6, 24, 50, and 75 hr had the highest sCOD removal efficiency (96%–98%) and lower removal (92%) at HRT 10 and 16 hr. sCOD concentrations in the electro‐bioreactors effluent were 79.1 ± 0.9, 173 ± 1.0, 177 ± 1.0, 27.05 ± 0.95, 58.95 ± 1.85, and 70.15 ± 0.35 mg L^−1^ at the HRT of 6, 10, 16, 24, 50 and 75 hr, respectively.

The removal efficiency of PO_4_^3−^–P in the control bioreactor improved considerably at higher HRT, with 32%–42% removal at lower HRT's of 6, 10, and 16 hr compared with 94%–98% removal at HRT's of 24, 50 and 75 hr (Figure 2b). PO_4_^3−^–P concentrations in the control bioreactor effluent were 4.1 ± 0.1, 4.5 ± 0.0, 4.8 ± 0.0, 0.3 ± 0.0, 0.07 ± 0.06 and 0.4 ± 0.0 mg L^−1^ at HRT of 6, 10, 16, 24, 50 and 75 hr, respectively. High removal efficiencies of PO_4_^3−^–P ranging from 96% to 99% were measured in the electro‐bioreactors at all HRT tested. Electro‐bioreactors operated at HRT of 6, 10 and 16 had 99% removal efficiency with PO_4_^3−^–P concentrations in the effluent of 0.02 ± 0.0, 0.003 ± 0.001 and 0.04 ± 0.0 mg L^−1^, respectively.

The removal efficiency of NH_4_^+^–N in the control bioreactors increased from 61.7% at HRT of 6 hr to 99% at the other tested HRT's, while the removal efficiency observed in electro‐bioreactors was close to 100% at all HRT tested (Figure 2c). The effluent concentrations of NO_3_^−^–N in the control bioreactors were 6.4 ± 0.2, 5.6 ± 0.1, 0.4 ± 0.3, 1.6 ± 0.8, 8.3 ± 0.4 and 5.1 ± 0.7 mg L^−1^ at HRT of 6, 10, 16, 24, 50 and 75 hr, respectively (Figure 2d). The corresponding measured electro‐bioreactor effluent NO_3_^−^–N concentrations were 10.3 ± 0.4, 12.2 ± 0.3, 0.2 ± 0.0, 0.2 ± 0.0, 0.9 ± 0.1 and 4.1 mg L^−1^ at HRT of 6, 10, 16, 24, 50 and 75 hr, respectively.

### 3.2 Effect of HRT on total bacterial counts (TBC in CFU mL^−1^)

Bacteria form an essential component of the microbial community representing the activated sludge. Their total count fluctuations can reflect the efficiency of the sludge to process the organics found in wastewaters. It is well understood that using the plate count method to enumerate microbes in environmental samples vastly underestimates numbers because the vast majority of microbes do not grow in laboratory cultures (Madigan et al., 2014). Despite this, it has been extensively used to compare samples and represents one way of measuring how a variable affects viability of microbes in a sample. The plate count method was used to determine bacterial counts in the various bioreactor configurations we tested. Bacterial counts in the control bioreactors increased as HRT was increased and peaked at 11.8 × 10^6^ CFU mL^−1^ at HRT of 50 hr, then dropped severely at HRT of 75 hr (2.5 ± 0.1 × 10^6^ CFU mL^−1^). (Figure 2e). This means that dilution, as the main consequence of high hydraulic load, has affected the microbial community size which is the decrease in sludge biomass and disaggregation of larger flocs. Interestingly, bacterial counts in electro‐bioreactors were significantly increased compared to the controls at HRT's of 24, 50 and 75 hr (68.3 ± 0.4 × 10^6^ vs 3.5 ± 0.1 × 10^6^, 23.9 ± 0.1 × 10^6^ vs 11.8 ± 0.1 × 10^6^, and 3.6 ± 0.2 × 10^6^ vs 2.5 ± 0.1 × 10^6^ CFU mL^−1^, respectively). The bacterial counts in control and electro‐bioreactors were similar at HRT's of 6, 10 and 16 hr. This means that an electric field at current density of 3 Am^−2^ stimulated bacterial growth at higher HRT, which we have previously reported (Zeyoudi et al., 2015), but not at lower HRT's. These findings assume the high removal efficiency of sCOD, PO_4_^3−^–P and NH_4_^+^–N at different HRT tested could be due to electrokinetic process taken together with the high density of bacterial community present in the electro‐bioreactors (Henze, 2008).

### 3.3 Overall bacterial diversity in control bioreactors and electro‐bioreactors

**Figure 3:**
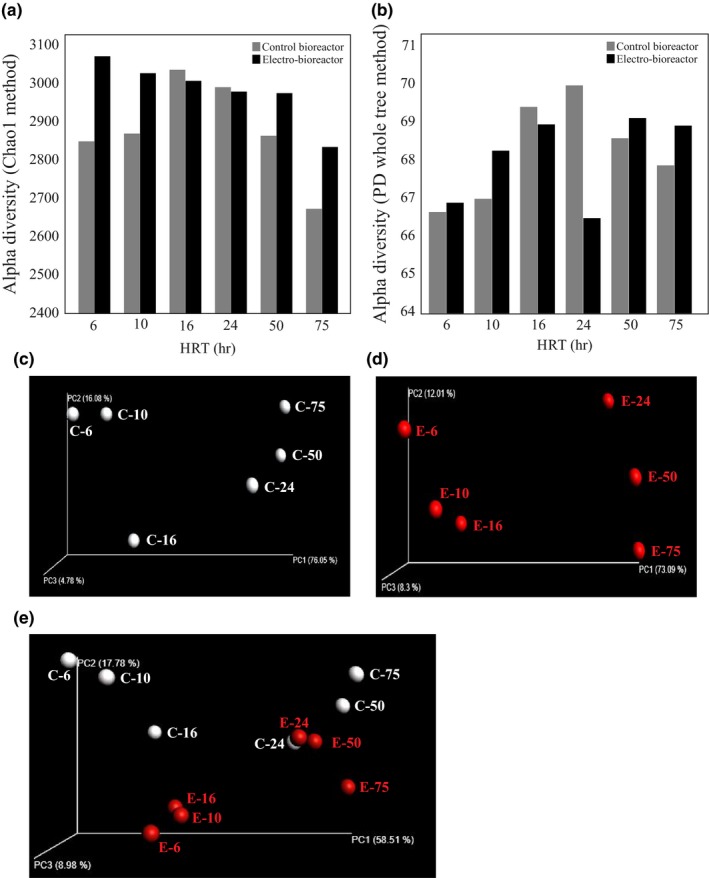
Alpha (α) diversity richness calculations using (a) Chao1 and (b) PD whole tree methods. Three‐dimensional principal coordinate analysis (PCoA) plot showing the bacterial community variations present in (c) control bioreactors (C‐ “HRT hours”), (d) Electro‐bioreactors (E‐”HRT hours”), and (e) both control and electro‐bioreactors under different HRTs

Microbial community structure and function influences the performance of wastewater reactors, which in turn has an impact on the treated water quality (Henze, 2008; Tchobanoglous et al., 2003). To compare the bacterial communities in bioreactors and electro‐bioreactors subjected to different HRT conditions, we performed (α) diversity analysis on each sample and (β) diversity analysis across the samples. α diversity expresses the diversity of a population within a system; a community will have a higher α diversity when there is a higher number of unrelated species within the same sample (Jost, 2007). α diversity was assessed, using the *Chao1* index and *Phylogenetic Diversity* (PD_whole_tree) index (Faith & Baker, 2006) (Figure 3a). These measurements indicate different α diversities in control bioreactor samples under different HRTs. Specifically, control bioreactors operated at HRT of 6, 10, 50, and 75 hr had lower α diversity value than control bioreactors operated at HRT of 16 and 24 hr. This finding was similar to bacterial diversity in a moving bed biofilm reactor (MBBR) under different HRTs (5, 10 and 15 hr) reported by (Calderón et al., 2012). Additionally, electro‐bioreactors with HRTs of 6, 10, 16 and 50 hr had higher α diversity than electro‐bioreactors with HRTs of 24 and 75 hr (Figure 3b). Electro‐bioreactor at HRT of 6 hr had the highest α diversity *Chao1* index across the 12 samples. This indicates that the electric field applied at current density of 3 Am^−2^ produced a series of electrochemical reactions (electron donors and acceptors) which in other words energy in integration with carbon and nutrients supplied from the synthetic wastewater resulted in broadening the diversity of bacterial community. Correlation analysis using ANOVA statistical test between α diversity and physicochemical and operational variables showed that bacterial α diversity was not significantly correlated with sCOD, but was significantly correlated with PO_4_^3−^–P, NH_4_^+^–N and NO_3_^−^–N (coefficient of 0.10, *p*‐value < .05). Additionally, other physiochemical such as temperature, pH and electrical conductivity had no statistically significant (*p*‐value > .05) correlation with α diversity matrices indicating that these parameters may have little impact on bacterial diversity within these samples and agrees with previous reports (Ju & Zhang, 2014).

To investigate what physiochemical and operational parameters best correlated with the variability in bacterial abundance and diversity from each sample, A BIO‐ENV trend and Spearman's rank correlation analysis (using QIIME v 1.9.0) was performed (that is β‐diversity). This type of analysis calculates a bacterial community difference distance matrix and compares to Euclidian distance matrixes for each of the other measured variables (such as temperature, pH, ammonia content, etc.). The variables that best explained the weighted Unifrac distance between all OTUs were best correlated with incorporation of HRT, pH, PO_4_^3−^–P, NH_4_^+^–N, NO_3_^—^N, and the application of an electrical current (correlation = 0.51, 0.32, 0.44, 0.26, 0.11 and 0.09, respectively). Principle Coordinate Analysis (PCoA) plots were generated by Qiime software using phylogeny‐based‐weighted Unifrac distance analysis resulting from β diversity calculations in order to compare the bacterial communities under different HRT (Ju & Zhang, 2014) (Figure 3c, d, and e). When looking only at the control reactors or electro‐bioreactors alone, microbial communities cluster together on two distinct sides of the curve (Figure 3d, e). As can be seen in these PCoA plots, microbial communities from reactors operated at HRT of 6, 10, and 16 hr cluster on the left side of the graph, while samples from reactors operated at HRT of 24, 50, and 75 hr on the right side. Therefore, it can be said the quite distinct microbial communities develop when short (6, 10, 16 hr) vs long (24, 50, 75 hr) HRT are used in the absence and presence of an electric current. Indeed, it has previously been reported that microbial communities in various types of bioreactors are dependent on the HRT used (Klimiuk & Kulikowska, 2006; Zhang et al., 2015). In Figure 3e, we generated a PCoA plot to compare microbial community samples in the control bioreactor vs the electro‐bioreactor running at the same HRT. The plot shows that microbial communities in control and electro‐bioreactors are different at low HRTs (6, 10, and 16 hr) and converge to almost identical communities at an HRT of 24 hr, then diverge again at the longer HRT of 50 and 75 hr (Figure 3e).

### 3.4 Phylogeny and abundance of overall bacterial communities

**Figure 4:**
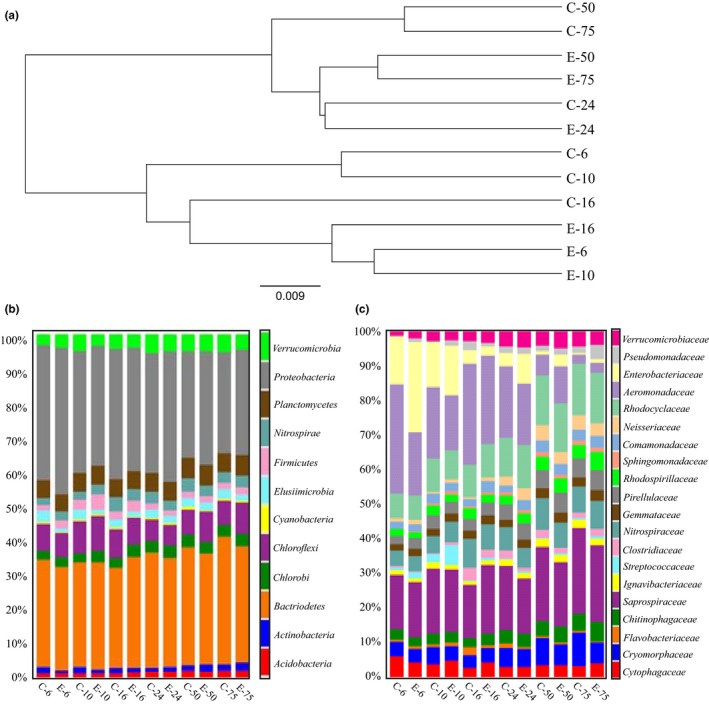
(a) Beta (β)‐diversity using UPGMA clustering analysis using weighted Unifrac. Phylogeny of the bacterial communities in the control bioreactor and electro‐bioreactors at (b) phylum level, and (c) family level

Raw Illumina MiSeq sequencing data was analyzed, using QIIME for the 12 samples discussed here. Assigning sequences to different OTUs resulted in a total of 213,738 OTUs with a minimum sequence depth of 66,886 per sample and a table density of 0.100. 118,628 of the OTUs could not be assigned to any bacterial phylum. Furthermore, after trimming and filtering according to the criteria mentioned in the materials and methods section, the OTU biom table resulted in 3,402 observed OTUs and a table density of 0.771 with a minimum sequencing depth of 34,273.0 was obtained. Rarefaction analysis of our sequencing data indicated that the sequencing depth we achieved per sample was sufficient to uncover low abundance species as rarefaction plots of reads vs OTU's leveled off. β‐diversity analysis using UPGMA clustering revealed that the bacterial communities in the 12 samples could be clustered into two main groups containing: (i) control bioreactor and electro‐bioreactors samples at long HRT of 24, 50 and 75 hr. (ii) control bioreactors and electro‐bioreactors at short HRT of 6, 10 and 16 hr. (Figure 4a). Once separated into the two main branches, the presence or absence of an electric current was the next most important factor. For example, microbial communities from control reactors at HRT of 6, 10 and 16 hr clustered together on the short HRT main branch, while microbial communities from electro‐bioreactors at HRT of 6, 10 and 16 hr clustered together on the short HRT main branch (Figure 4a). This was also the case on the long HRT main branch with the exception at HRT of 24 hr, where the control and electro‐bioreactor microbial communities clustered closer together. This is also reflected in the PCoA plots in Figure 3e. This means that short HRT had a crucial impact on microbial community structure present in electro‐bioreactors running under electric field of current density 3 Am^−2^. Phylogenetic analysis of the microbial communities present in the different reactors tested showed that *Proteobacteria* accounted for the largest fraction (35.3% average abundance across all samples) which is consistent with previous work (González‐Martínez et al., 2013; Klimiuk & Kulikowska, 2006), followed by *Bacteroidetes* (32.3%), *Chloroflexi* (7.9%), *Planctomycetes* (5.5%), *Verrucomicrobia* (4.5%), *Chlorobi* (3.3%), *Nitrospirae* (3.1%) and *Firmicutes* (2.3%) (Figure 4b). Generally, *Proteobacteria* abundance was correlated with the electric current density, as relative abundances observed in electro‐bioreactors operated at HRT of 6, 24, 50, and 75 hr were 42.6, 37.9, 33.0, and 30.9%, respectively, while lower abundances was observed in the control bioreactors run at the same HRTs (39.1, 34.7, 31.1 and 29.4%, respectively). This correlation did not hold true (show the same trend) in the reactors run at HRT of 10 and 16 hr (34.8 and 36.0 in the electro‐bioreactors compared to 35.5 and 38.0%, in the control bioreactors). *Bacteroidetes* was inversely correlated with the electric current density as it found to be more abundant in control reactors operated at HRT of 6, 24, 50 and 75 hr (31.3, 33.6, 34.4, and 37.5%, respectively), than in electro‐bioreactors operated at HRT of 6, 24, 50, and 75 hr (30.3, 32.0, 32.3 and 33.8%, respectively). Both electro‐bioreactors operated at HRT of 10 and 16 hr had higher *Bacteroidetes* relative abundance (31.1 and 32.2%, respectively) than in control bioreactors operated at HRT of 10 and 16 hr (30.3 and 29.2%, respectively). It was observed there is a competition between bacterial species under those two phyla in a way that if one is present in high abundance the other will appear in lower relative abundance. This means that there is a minor shift in bacterial communities from *Bacteroidetes* to *Proteobacteria* which could be due to the operational conditions of the system that increase the biological activity of a subpopulation to another (Ibarbalz et al., 2013; Thenmozhi, Uma, & Meenambal, 2015). *Firmicutes* were found in higher relative abundances in all electro‐bioreactors than control bioreactors at all HRTs tested except for 24 hr (Ahn et al., 2007; Hu, Wang, Wen, & Xia, 2012; Nguyen, Le, Hansen, Nielsen, & Nielsen, 2011; Nielsen et al., 2010; Wang, Hu, Xia, Wen, & Ding, 2012). Our phylogenetic analysis also indicated that there were 49 bacterial families present in all samples. The 20 families with higher than 0.5% average abundance across all samples are shown in Figure 4c. *Saprospiraceae* (18.7), *Aeromonadaceae* (16.8%), *Rhodocyclaceae* (11.1%), *Enterobacteriaceae* (7.6%), *Nitrospiraceae* (16.7%) were the top five abundant families across all samples. Our results showed that highest abundance of *Rhodocyclaceae* was observed in bioreactors operating at HRT of 75 hr (14.8 and 14.6% in the control and electro‐bioreactors). *Nitrospiraceae* was observed to be in high abundance when HRT of 16 and 50 hr were used (8.3 and 9.0% in the control reactor versus 7.2 and 8.2% in the electro‐bioreactors, respectively). *Saprospiraceae* reached highest abundance at HRT of 75 hr (24.9 and 22.1% in the control and electro‐bioreactor, respectively). Additionally, *Enterobacteriaceae*,* Verrucomicrobiaceae*,* Neisseriaceae*,* Pseudomonadaceae* and *Sphingomonadaceae* were present in electro‐bioreactors operated at HRT of 6 hr with higher relative abundance (26.1, 2.1, 1.1, 0.9 and 0.5%, respectively) than in the control bioreactors (13.8, 1.3, 0.8, 0.4 and 0.2%, respectively). Therefore, these bacterial families favored electro‐bioreactors subjected to an electric current density of 3 Am^−2^ operated at HRT of 6 hr were affiliated with better biological nutrient removal (Cydzik‐Kwiatkowska & Zielińska, 2016; Lee et al., 2014; Nielsen et al., 2010; Valentín‐Vargas, Toro‐Labrador, & Massol‐Deyá, 2012) (Figure 2a, b, c and d).

### 3.5 Comparison of functional bacterial genera response to short and long HRTs in control bioreactors and electro‐bioreactors

**Figure 5:**
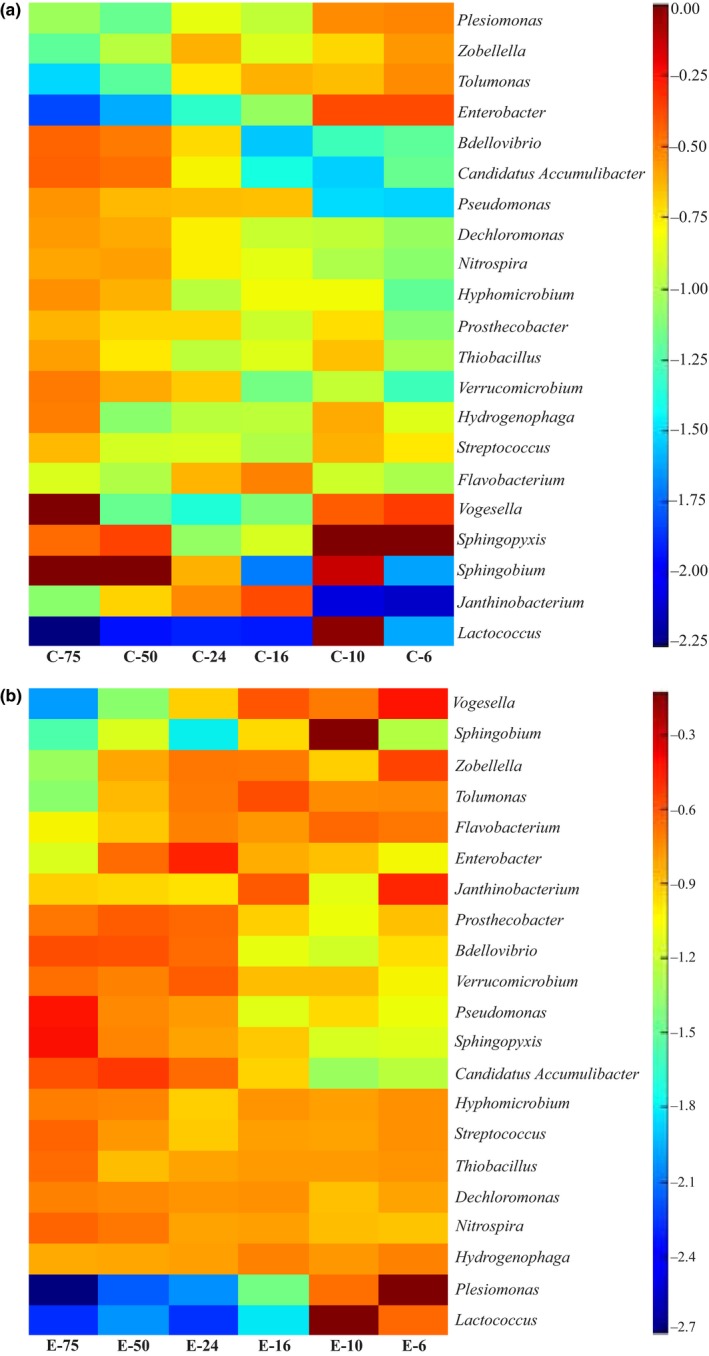
Heat map of functional genera in (a) control bioreactors and (b) electro‐bioreactors under different HRTs

To provide a clearer picture of the shifts we observed in the subpopulations of functionally relevant bacteria in our different reactors, two heat maps were constructed illustrating these differences (Figure 5a and b). To provide a more simplified comparison between the functional bacterial genera under different HRT conditions, we classified the HRT conditions into short HRT (6, 10 and 16 hr) as shown in Table 3 and long HRT (24, 50 and 75 hr) as shown in Table 4. The trends observed regarding the abundance of these functionally relevant genera fell into one of the following eight categories. The first category are bacterial genera that were observed to increase as HRT increased regardless of the presence or absence of a current and includes *Nitrospira*,* Dechloromonas, Pseudomonas and Bdellovibrio*. In contrast, the second category is represented by genera that decreased as HRT increased in the presence or absence of an electrical current, and include *Enterobacter*,* Plesiomonas, Zobellella, Tolumonas, Vogesella*, and *Lactococcus*. The third category contains bacterial general that always or almost always increased in the electro‐bioreactors compared to the controls at all HRTs, and include *Lactococcus*,* Streptococcus*,* Hyphomicrobium, Sphingopyxis, Sphingobium, Hydrogenophaga, Janthinobacterium, Thiobacillus, Vogesella, Dechloromonas, Zobellella, Plesiomonas, Pseudomonas*, and *Verrucomicrobium*. The fourth category contains bacterial genera that always or almost always decrease in the electro‐bioreactors compared to the controls at all HRTs, and include *Enterobacter* and *Tolumonas*. The fifth category contains bacterial genera that increase in abundance at Short but not long HRTs in electro‐bioreactors compared to controls, and include *Nitrospira*,* Bdellovibrio*, and *Candidatus Accumulibacter*. The sixth category contains bacterial genera that increase in abundance at long but not short (more than at short HRT) HRTs in electro‐bioreactors compared to controls, and include *Pseudomonas*,* Streptococcus, Enterobacter*, and *Tolumonas*. The seventh category contains bacterial genera that decrease in abundance at short but not long HRTs in electro‐bioreactors compared to controls, and include *Enterobacter* and *Tolumonas*. The eighth category contains bacterial genera that decrease in abundance at long but not short HRTs in electro‐bioreactors compared to controls, and include *Nitrospira*,* Bdellovibrio*, and *Candidatus Accumulibacter*.

**Table 3:** OTU counts of various functional bacterial genera in control bioreactor (C) and electro‐bioreactors (E) operated at short HRTs (6, 10, and 16 hr) and their corresponding role in nutrient and pollutant removal

Role	Genus	C – 6	E – 6	C ‐ 10	E ‐ 10	C– 16	E – 16	OTUs
*High abundance*								
P‐removal	*Dechloromonas*	174	303	206	262	283	361	29
	*Candidatus Accumulibacter*	39	28	17	23	29	56	7
Total		213	331	223	285	312	417	
N‐removal – AOB	*Nitrospira*	1144	1343	1298	1455	2444	1765	44
BOD and NH_4_^+^‐N removal	*Janthinobacterium*	2	482	2	117	130	358	28
Total		1146	1825	1300	1572	2574	2123	‐
Degrading sCOD ‐ Dairy wastewater treatment plants	*Lactococcus*	12	362	405	1238	7	27	14
	*Flavobacterium*	172	228	196	263	640	193	30
	*Vogesella*	38	107	29	59	8	74	3
Total		222	697	630	1560	655	294	‐
Denitrifiers ‐ sCOD reduction ‐ Bioremediation	*Pseudomonas*	91	270	87	356	755	260	39
Glucose fermenters ‐ Bioaugmentation	*Streptococcus*	158	162	197	150	117	154	9
Beneficial bacteria	*Bdellovibrio*	243	285	184	196	142	230	20
Antibiotic resistance	*Enterobacter*	1106	75	1027	110	287	124	10
*Low abundance*								
Denitrifying‐methylotroph‐ able to degrade xenobiotic products	*Hyphomicrobium*	26	93	59	88	79	96	6
Denitrifiers ‐ Hydrogen oxidizing bacteria	*Hydrogenophaga*	22	32	36	29	22	33	8
Industrial production of polyhydroxybutyrate	*Zobellella*	46	51	30	22	28	38	1
Bisphenol A	*Sphigobium*	3	6	81	79	3	12	2
Sulfur	*Thiobacillus*	49	83	97	82	83	82	2
Adsorb cadmium ions (Cd^2+^)	*Plesiomonas*	17	540	15	163	8	28	13

**Table 4:** OTU counts of various functional bacterial genera in control bioreactor (C) and electro‐bioreactors (E) operated at long HRTs (24, 50, and 75 hr) and their corresponding role in nutrient and pollutant removal

Role	Genus	C‐24	E‐24	C‐50	E‐50	C ‐75	E‐75	OTUs
*High abundance*								
P‐removal	*Dechloromonas*	274	194	257	276	196	262	29
	*Candidatus Accumulibacter*	82	59	109	109	85	83	7
Total		356	253	366	385	281	345	‐
N‐removal ‐ AOB	*Nitrospira*	1952	944	1952	1627	1324	1665	44
BOD and NH_4_^+^–N removal	*Janthinobacterium*	63	84	27	120	8	114	28
Total		2015	1028	1979	1747	1332	1779	‐
Degrading sCOD ‐ Dairy wastewater	*Lactococcus*	5	5	3	12	1	6	14
	*Flavobacterium*	335	122	97	105	89	72	30
	*Vogesella*	3	19	3	9	0	2	3
Total		343	146	103	126	90	80	‐
Denitrifiers ‐ sCOD reduction ‐ Bioremediation	*Pseudomonas*	528	294	350	432	303	838	39
Glucose fermenters ‐ Bioaugmentation	*Streptococcus*	103	64	65	119	79	150	9
Beneficial bacteria	*Bdellovibrio*	626	329	680	520	544	486	20
Antibiotic resistance	*Enterobacter*	103	163	37	139	16	43	10
*Low abundance*								
Denitrifying‐methylotroph‐ able to degrade xenobiotic products	*Hyphomicrobium*	38	36	52	76	44	72	6
Denitrifiers ‐ Hydrogen oxidizing bacteria	*Hydrogenophaga*	15	15	7	19	19	17	8
Industrial production of polyhydroxybutyrate	*Zobellella*	34	21	10	21	4	6	1
Adsorb cadmium ions (Cd^2+^)	*Plesiomonas*	7	4	2	4	2	1	13
Bisphenol A	*Sphigobium*	24	1	0	6	0	2	2
Sulfur	*Thiobacillus*	47	43	46	48	49	74	2

**Figure 6:**
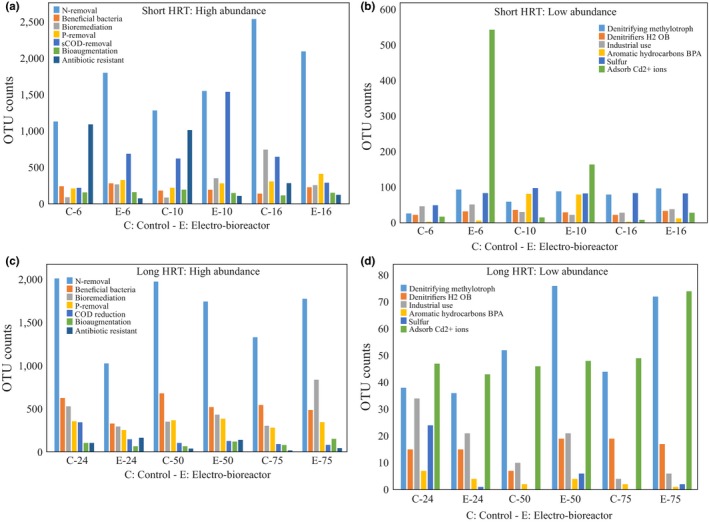
Graph indicating differences in the relative abundance represented in OTU counts of functional bacterial genera between control bioreactors and electro‐bioreactors: (a) high abundant bacterial genera at short HRTs, (b) low abundant bacterial genera at short HRTs, (c) high abundant bacterial genera at long HRTs, and (d) low abundant bacterial genera at long HRTs

At short HRT, there was a noticeable higher relative abundance of functional bacteria in reactors operated at short HRTs known to be associated with N‐removal, such as *Nitrospira* and *Janthinobacterium sp*., P‐removal, such as *Dechloromonas sp*. and sCOD reduction, such as *Lactococcus, Flavobacterium*, and *Vogesella* (Figure 6a and b). This indicates that growth and enrichment of functional bacterial species occurred in electro‐bioreactors operated at short HRTs compared to the control (Table 3), resulting in better biological nutrient removal. This confirms that the metabolic activity of these species increased under the impact of electric field at current density 3 Am^−2^ and short HRT. Other species which are functionally important such as *Pseudomonas sp.,* a denitrifying species which has been previously confirmed to utilize different organic compounds and has been directly linked to sCOD removal in microbial fuel cells (Majumder et al., 2014), had higher observed OTU counts in electro‐bioreactors operated at short HRT when compared to the control. *Pseudomonas sp*. has been reported to also be involved in bioremediation in municipal wastewater treatment (Wasi, Tabrez, & Ahmad, 2013). Additionally, an observed increase in OTU counts for microorganisms such as *Hyphomicrobium sp*. and *Plesiomonas sp*. in electro‐bioreactors operated at short HRT than in control bioreactors (Table 3).

*Hyphomicrobium* is a denitrifying methylotroph, meaning that they reduce nitrate and use methane as a carbon and energy source. *Plesiomonas sp*. is recently discovered to adsorb cadmium ions which can be applied in wastewater treatment plants in the future (Xue, Qi, Li, & Liu, 2016). The presence of an active population of fermenting bacteria that supply substrates to other functional groups is therefore of fundamental importance for efficient N and P removal carried out by the activated sludge process in wastewater treatment systems. Fermenting microorganisms use an internally balanced redox process in which the organic substrate becomes both oxidized and reduced. *Streptococcus*, a glucose fermenting species, was found to be in slightly higher abundance in electro‐bioreactors operated at short HRT compared to control bioreactors (Table 3). There was a slight increase in relative abundance of certain bacterial species in electro‐bioreactors operated at short HRT which is originally present in low abundance as shown in Figure 6b, such as sulfur reducing bacteria (*Thiobacillus*), hydrogen oxidizing bacteria (*Hydrogenophaga*), recently discovered species to be associated with industrial production of polyhydroxybutyrate (*Zobellella*) (Ibrahim & Steinbüchel, 2010) and micropollutant removing bacteria such as aromatic hydrocarbons and BPA (*Sphingobium*) (Sasaki, Maki, Oshiman, Matsumura, & Tsuchido, 2005) (Table 3).

Interestingly, there was an observed increase in the relative abundance of a known beneficial bacteria *Bdellovibrio sp*. in electro‐bioreactors operated at short HRTs compared to control bioreactors (Table 3). *Bdellovibrio* and like organisms (BALOs) are small, predatory *Deltaproteobacteria* that prey on other gram‐negative pathogens. Many authors have unfolded the possible use of BALOs as biological control agents in environmental as well as medical microbiological settings. They are found strongly associated with natural biofilms and recent studies have shown that effective predation occurs in these naturally occurring bacterial communities. Accordingly, recent researches aim to present the evolution toward applying *Bdellovibrio* as an antibacterial agent to deal with oral infections, general medical conditions, environmental, and industrial issues (El‐Shanshoury, Abo‐Amer, & Alzahrani, 2016; Harini, Ajila, & Hegde, 2013).

In contrast to the genera mentioned above in which application of current enhanced their growth at short HRT's, relative abundance of the *Enterobacter sp*. decreased in electro‐bioreactors at short HRTs. *Enterobacter sp*. are known as antibiotic resistant bacteria (Meyer, Saunders, & Blackall, 2006; Nielsen et al., 2010), and their presence or depletion is important in determining the efficiency of a wastewater treatment technology and effluent quality (Espigares, Bueno, Espigares, & Gálvez, 2006; Filipkowska, 2003). At long HRT, bacterial communities associated with N‐removal such as *Nitrospira sp*. favored control bioreactors than electro‐bioreactors as shown in Figure 6c and d, as their relative abundance was higher than in control bioreactors operated at HRT of 75 hr as shown in Table 4. The results for HRT of 24 hr agreed with previously reported research studying the biodegradation of trichloroethylene wastewater and anaerobic bacterial community in the UASB reactor (Zhang et al., 2015). *Dechloromonas*,* Pseudomonas*,* Sphingobium*, and *Bdellovibrio sp*. abundances showed the same trend which means that these bacterial communities did not favor conditions of applying electric field of current density of 3 Am^−2^ at HRT of 24 and 50 hr. This could be due to the lower dissolved oxygen (5.0 and 3.9 mg L^−1^) in electro‐bioreactors operated at HRT of 24 and 50 hr in combination with lower organic loading lead to decrease in the metabolic activity of bacterial species present in electro‐bioreactors operated at HRT of 24 and 50 hr. On the contrary, *Streptococcus sp*. favored long HRT than short HRT as it was observed to double in counts in electro‐bioreactors operated at HRT of 50 and 75 hr (119 and 150 OTU counts, respectively) to control bioreactors of 50 and 75 hr (65 and 79 OTU counts, respectively). This could illustrate the high removal efficiency of N, P and sCOD as shown in Figure 2. Our results indicated that most of the bacterial communities present in electro‐bioreactors were stimulated and favored conditions of short HRT of 6, 10 hr and long HRT of 75 hr while in control bioreactors it favored HRT of 24 hr. This means that HRT in control bioreactors and electro‐bioreactors plays a crucial role in reactor performance and in turn effluent quality. Also, lower HRTs would reduce the volumes of the electro‐bioreactors, which in turn will have great economic savings.

## 4 DISCUSSION

### 4.1 Reactor performance and physiochemical parameters under different HRTs

The highest differences in sCOD removal between the control bioreactor and the electro‐bioreactor were at the ones operated under HRT of 6, 24, 50, and 75 hr. The decrease in sCOD removal efficiency in control bioreactors operated at short HRT could be related to the shorter contact time between the activated sludge and organic matter. In addition, the decrease in sCOD removal efficiency might also be due to the fact that some bacteria were washed out from the sequencing batch reactor when HRT was less than the facultative bacteria‐generation time (Kapdan, 2005; Yang et al., 2006). However, in electro‐bioreactors operated at HRT of 6 hr, there was an increase in sCOD removal efficiency which is due to electrocoagulation process resulted from the impact of electric field (Giwa & Hasan, 2015; Hosseinzadeh, Bidhendi, Torabian, Mehrdadi, & Pourabdullah, 2015).

The high removal of phosphorus in electro‐bioreactors at all HRTs is not only attributed to biodegradation (via precipitation in the sludge) but also to the electro‐deposition phenomenon through which phosphorus ions tend to deposit on the surface of the electrodes, mainly on the cathode, as previously reported (Giwa & Hasan, 2015; Ioan & Robescu, 2015; Klimiuk & Kulikowska, 2006).

In all reactors, it has been observed that there is unbalanced ammonium oxidation‐to‐nitrate and ammonium removal. These results agree with previous studies which assumed that the lack of balance between ammonium amount and nitrate formed in control bioreactors and electro‐bioreactors could be due to partial nitrification, denitrification in aerobic conditions (Klimiuk & Kulikowska, 2006). It has also been reported that NH_4_^+^‐N removal in a single, aerated reactor is caused by complete autotrophic nitrogen removal over nitrite, known as the CANON process (Peng, Wu, Yu, Ai, & Fu, 2013). The reported results here show that electro‐bioreactors operated at low HRT of 6, 10, and 16 hr enhanced reactor performance in removing both PO_4_^3−^–P and NH_4_^+^–N, with very low measured concentrations in the effluent which did not exceed ≃0.01 and 0.05 mg L^−1^, respectively. Taken together with results previously reported by our group (Zeyoudi et al., 2015), this confirms that optimizing current density and HRT‐operating parameters in an electro‐bioreactor enhances nutrient removal efficiency, resulting in cleaner effluent and high water quality.

## 5 FUNCTIONAL BACTERIA IN CONTROL AND ELECTRO‐BIOREACTORS IN LINKAGE TO BIOLOGICAL NUTRIENT REMOVAL

HRT is considered as one of the most important operating parameters affecting the performance and microbial community of the bioreactor (González‐Martínez et al., 2013; Ioan & Robescu, 2015; Wang et al., 2009, 2015; Zhang et al., 2015). P‐removal in electro‐bioreactors occurs due to electrocoagulation process (Giwa & Hasan, 2015) in addition to electric current stimulation effects on bacterial communities affiliated with P‐removal. As shown in Figure 2b, high removal efficiency of PO_4_^3−^–P (97%–99%) was observed in all reactors operated at higher HRTs. Interestingly, higher HRT's were also associated with high OTU counts for *Dechloromonas sp*. (Figure 5) in all reactors. Furthermore, relative abundance of *Dechloromonas* were high even at low HRT's in electro‐bioreactors compared to the control (Figure 5); these electro‐bioreactors at low HRTs were also very efficient at depleting PO_4_^3−^–P compared to controls. *Dechloromonas* are known to oxidize benzene with nitrate serving as the electron acceptors (Cydzik‐Kwiatkowska & Zielińska, 2016). This genus can reduce perchlorate and is also frequently reported as a Polyphosphate accumulating organisms (PAO) in biological wastewater treatment systems (Kong, Xia, Nielsen, & Nielsen, 2007), and thus likely contributes to denitrification and phosphorous removal in the reactors tested. Taken together, our data indicates that one of the mechanisms by which adding an electric current contributes to PO_4_^3−^–P removal is through the enhancement of *Dechloromonas* species growth at low HRT's typically used in industrial settings. Nitrifying bacteria such as *Nitrospira sp*. were observed to be stimulated in electro‐bioreactors at HRT of 6, 10 and 16 hr (21.5, 22.3 and 26.9%, respectively) compared to control bioreactors (14.0, 17.4 and 24.7%, respectively). *Nitrospira* have been recognized as the numerically dominant nitrite‐oxidizing bacterial genus primarily responsible for the second step of aerobic nitrification (Fujitani, Aoi, & Tsuneda, 2013). Recent findings by (Daims et al., 2015) reported that the genome of the *Chemolithoautotrophic* nitrifying bacterium encodes the pathways both for ammonia and nitrite oxidation, which are concomitantly activated during growth by ammonia oxidation to nitrate. These findings point to completely nitrifying *Nitrospira* as key components of nitrogen‐cycling microbial communities (Daims et al., 2015; Juretschko et al., 1998). Indeed, the only reactor with poor ammonia removal was the control reactor at HRT of 6 hr, and this reactor had the lowest *Nitrospira* counts (Figures 2c and 5). All the other reactors containing higher relative abundance of *Nitrospira* sp. were associated with higher N‐removal efficiency (Figures 2c and 5).

sCOD indicates organic pollutants in the wastewater. Bacteria oxidizes organic compounds in the wastewater for their growth and metabolism (Henze, 2008). Therefore, the efficiency of wastewater treatment can be also assessed by measuring sCOD removal. As mentioned earlier, sCOD removal efficiency was high in all electro‐bioreactors compared to the control bioreactors (Figure 2a). Here, sCOD removal efficiency in the control bioreactors ranged between 92% and 94% at all HRT tested, while sCOD removal reached 96% to 98% in electro‐bioreactors at HRTs of 6, 24, 50, and 75 hr (Figure 2a). *Pseudomonas* and *Flavobacterium* have been previously reported that they can remove sCOD (Abdel‐Raouf, Al‐Homaidan, & Ibraheem, 2012; Nasr, 2010). Additionally, there was an observed high removal of sCOD in electro‐bioreactors operated at HRT of 6 hr which could be due to the electric current impact resulting in stimulating the growth of bacterial communities associated with sCOD reduction such as *Lactococcus, Pseudomonas* and *Flavobacterium* (Lee et al., 2014; Nielsen, Nguyen, Meyer, & Nielsen, 2012; Park et al., 2007). Our results showed that there was a higher relative abundance of those genera in electro‐bioreactors operated at HRT of 6 hr (5.8, 4.3 and 3.7%, respectively) than in control bioreactors (0.1, 1.1, and 2.1%, respectively). Indeed, species of the genus *Lactococcus* could produce lactate by fermentation of glucose. The ability of *Lactococcus* to degrade sCOD in trichloroethylene wastewater has been previously reported as well (Zhang et al., 2015). Taken together, we propose that a dual mechanism of sCOD removal is occurring in the electro‐bioreactor at the low HRT of 6 hr: An electrocoagulation process where organic compounds form coagulates that precipitate out of the wastewater in addition to biodegradation process where microorganisms stimulated by the current oxidize or sequester these organic compounds. This dual mechanism most likely holds true for all the other nutrients we measured in these experiments. Finally, this data represents the results that describe microbial communities that evolved when an initial sample of activated sludge is passed through a synthetic wastewater bioreactor under various operating parameters for 24 hr. The microbial communities that evolve if this was repeated with different starting material and real wastewater with different characteristics will most likely be different.

## 6 CONCLUSION

Results showed that electro‐bioreactors operated at CD of 3 Am^−2^ under different HRT conditions could effectively remove sCOD (96%–98%), PO_4_^3−^–P (96%–99%) and NH_4_^+^–N (99%) compared to control bioreactors at short HRTs. The relative abundances of functional bacterial genera varied depending on short and long HRT tested in both control bioreactors and electro‐bioreactors. We also discussed changes observed in the microbial population structure and how they potentially relate to reactor performance and effluent quality. These are the first results to describe effects of varying HRT on microbial community structure in wastewater electro‐bioreactors.

## CONFLICT OF INTEREST

Authors have no conflict of interest to declare.
